# Productive Behavior and Carcass Yield of Mexican Tropical Hairless Creole Pigs Fed Different Diets

**DOI:** 10.3390/ani15172583

**Published:** 2025-09-02

**Authors:** Adalberto Rosendo-Ponce, Carlos M. Becerril-Pérez, Alejandro Sánchez-Carrillo, Juan M. Vargas-Romero, Fredy Morales-Trejo, Lorena Luna-Rodríguez, Ramón Marcos Soto-Hernández, Luis M. Carrillo-López

**Affiliations:** 1 Posgrado en Agroecosistemas Tropicales, Colegio de Postgraduados Campus Veracruz, Km. 88.5 Carretera Federal Xalapa-Veracruz, Manlio Fabio Altamirano, Veracruz 91690, Mexico; arosendo@colpos.mx (A.R.-P.); color@colpos.mx (C.M.B.-P.); sanchez.alejandro@colpos.mx (A.S.-C.); fredy.morales@colpos.mx (F.M.-T.); 2 Posgrado en Ganadería, Colegio de Postgraduados, Campus Montecillo, Carretera Mexico-Texcoco Km. 36.5, Texcoco 56264, Mexico; 3 Departamento de Biología de la Reproducción, Universidad Autónoma Metropolitana, Unidad Iztapalapa, Av. San Rafael Atlixco 186, Leyes de Reforma 1ra Secc., Iztapalapa, Ciudad de México 09340, Mexico; jmvr@xanum.uam.mx (J.M.V.-R.); llunaro@xanum.uam.mx (L.L.-R.); 4 Posgrado en Botánica, Colegio de Postgraduados, Campus Montecillo, Carretera Mexico-Texcoco Km. 36.5, Texcoco 56264, Mexico; msoto@colpos.mx; 5 Secretaría de Ciencia, Humanidades, Tecnología e Innovación, Av. Insurgentes Sur 1582, Col. Crédito Constructor, Demarcación Territorial Benito Juárez, Ciudad de México 03940, Mexico

**Keywords:** *Sus scrofa* ssp. *domestica*, pen performance, primal cuts, fat, feed

## Abstract

The objective of this research was to determine the in-pen behavior and carcass yield of MCPs fed two different diets and slaughtered at two different live weights. The MCP biotype has a slow growth rate because they are fed with forage resources and locally available unconventional feeds. Sixteen castrated MCPs were used: eight pigs under 40 kg live weight and eight pigs over 40 kg. The diets were prepared with corn, soy, vitamins, and minerals, different protein levels, and the same energy content. Pigs fed the corn–soybean diet had a higher daily feed intake (500 g/d) and a significantly increased daily weight gain (160 g/d) compared to pigs fed on the corn-only diet, achieving slaughter weight in less time (4.33 times faster in pigs slaughtered at 40 kg live weight and 2.44 times faster for pigs slaughtered at 80 kg live weight). Regarding carcass yield, fat was 10% higher in pigs slaughtered at 80 kg compared to those at 40 kg. Soybean meal feeding improved the in-pen performance of MCPs but did not improve carcass yield. At higher slaughter weights, fat yield increased, but meat yield did not change.

## Simple Summary

In this study, diets were designed to improve pen performance and the yield of carcass and primal cuts of Mexican tropical hairless Creole pigs (MCPs). MCPs originate from pigs found in Celtic, Iberian, and Asian regions. Over time, they have developed resilience and adaptability to the conditions found in the coastal areas of Mexico through natural processes. Herds have a slow growth rate because they are fed with forage resources and locally available unconventional feedstuff. Scientific information to determine the adequate nutritional requirements for MCPs is lacking. Pigs fed soybean–corn feed consumed more feed and gained more weight per day, reducing the time in pen to reach slaughter weight. However, feed conversion and carcass yield were not improved. Feeding soybean–corn feed increased the yield of sirloin but not that of other primal cuts (ham, loin, shoulder, and rib). Slaughtered pigs with a higher live weight saw an increase in head and ham yield, and the carcass had a higher percentage of fat. The accumulation of fat in MCP allows for the preservation of traditional cuisine in the region.

## 1. Introduction

American pigs have descended from Iberian breeds. Currently, there is a wide variety of Creole pig phenotypes spread geographically from Mexico to the extreme south of Argentina [1]. The MCP is descended from pigs brought in the 16th century by the Castilian conquerors; specifically, the MCP was derived from Iberian-, Celtic-, and Neapolitan-type pigs (from Europe), with Asian-type pigs from China, Japan, or the Philippines [2]. The MCP is established in an endemic population located on the Pacific Ocean coasts of Mexico, in the states of Chiapas, Oaxaca, Jalisco, and Nayarit, and in the southeast of Mexico in the states of Quintana Roo, Yucatán, Campeche, Tabasco, and Veracruz [3]. Thus, Mexican hairless pigs are a variation of Creole pigs consisting of two genetic lines, that of the Gulf of Mexico and that of the Mexican Pacific coast [4]. The MCP, through a naturalization process, generated rusticity and adapted to climatic and ecological conditions distinct from those of its origin, with high temperatures and high relative humidity, different foods, and exposure to other pests and diseases [5].

The current MCP has suffered varying degrees of genetic erosion due to the introduction of commercial breeds oriented toward the production of lean meat [6]. MCP meat is used primarily for festive and religious occasions, with the sale of piglets and breeding stock being a source of income for families [7]. Small MCP herds are raised on plots of land in rural communities and fed with forage resources and unconventional foods or with agricultural products and by-products. The main ingredients are corn kernels (cob, whole kernels, cracked, and ground), dough, and crumbled tortillas and fruits, vegetables, leftover family meals, and forage grasses and legumes [8]. Furthermore, the MCP is of sociocultural importance in the study region (Veracruz, Mexico). Consequently, there have been a range of initiatives to promote biological conservation because the MCP is part of the worldview, customs, and gastronomy of social groups [9]. Unfortunately, economic problems in the region, population growth, and excessive urbanization have led to a fall in the number of animals [9]. Furthermore, scientific information with regard to the optimum nutritional requirements for MCPs is scarce. The formulation of optimal diets for MCP breeding in intensive production systems could support the subsistence economy in the region and provide a source of good-quality protein food, increasing the income obtained from the sale of animals and providing fertilizers for agriculture through manure composting and reinforcing a population’s identity [6]. The MCP is a local breed that guarantees food security for rural communities in the Gulf of Mexico. However, according to the FAO Domestic Animal Diversity Information System (DAD-IS 2024), it is considered to be locally adapted and endangered. Consequently, comprehensive studies are needed to ensure its conservation as a reservoir of genetic diversity [4].

The incorporation of corn and soybeans in conventional diets increases backfat and intramuscular fat in Creole pigs compared to commercial pigs (Landrace–Yorkshire). However, at the same age, both the carcass yield and the live weight of Creole pigs are significantly lower than those of commercial pigs [10]. Although fat accumulation is linearly associated with age and weight, in Creole pigs, this trend is much more pronounced due to their genetic constitution. For this reason, there has been a recent trend in the use of alternative protein sources in diet formulation. In Creole pigs, Dzib et al. [11] found that the inclusion of plant-based meals (*Moringa oleifera* and *Brosimum alicastrum*) in the diet of Creole pigs can reduce carcass fat and reduce feed and production costs in backyard or semi-intensive pig production systems [12]. Unlike what occurs in Creole pigs, in commercial pigs (Landrace × Large White), cereal-based diets lead to a greater carcass weight and greater daily weight gain compared to diets based on by-products [13]. Therefore, it is necessary to formulate specific diets in Creole pigs, which are slow growing, that lead to an increase in carcass yield and, at the same time, modulate the content and quantity of fat.

MCPs exhibit slow growth and a thick backfat layer at slaughter, with a steadily increasing rate of adipose tissue deposition, while the rate of muscle tissue deposition remains constant at a given age. As a result, feed conversion efficiency decreases, as adipose tissue is metabolically more expensive than muscle tissue [3]. However, body fat is distributed in intramuscular stores that provide unique characteristics in meat, such as a tender texture and improved flavor, desirable attributes for select cured meat products [4]. Given this situation, the objective of this study was to understand the productive behavior in the pen (in terms of feed consumption, weight gain, and feed conversion) and to evaluate the performance and characteristics of the carcass (head, meat, fat, and skin) and of the primary cuts of the MCP fed with two diets (corn base and corn–soybean) and a live weight at slaughter of 40 and 80 kg.

## 2. Materials and Methods

### 2.1. Location of This Study

**Figure 1:**
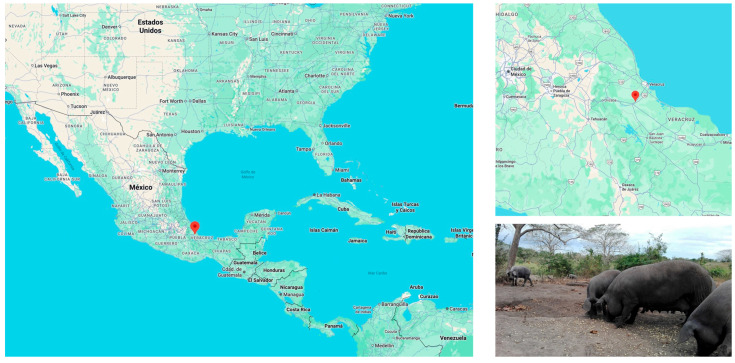
Map of research area (red mark).

This research was carried out in the town of La Capilla located on the Federal Highway Veracruz-Córdoba km 40, Municipality of Cotaxtla, Veracruz, Mexico, at 18°53′29″ N and 96°14′58″ W (Figure 1), with an altitude of 33 m above sea level and average annual precipitation and temperature of 1030 mm and 26 °C, respectively, climate Aw0 (w) (i′) g, warm subhumid, with rains in summer [14].

### 2.2. Experimental Development and Treatments

**Figure 2:**
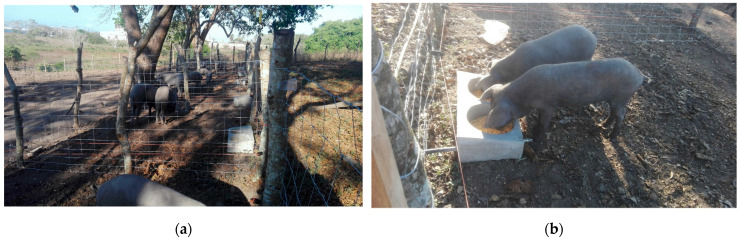
(**a**) Design of the pen for housing the Mexican Creole pigs (MCPs); (**b**) individual feeders for ad libitum feeding of MCPs.

Sixteen castrated MCPs were used, with eight pigs weighing less than 40 kg and eight pigs weighing over 40 kg, a body condition score greater than 2.5 (scale 1–5), and an average age of 8 months. The scale refers to the evaluation criteria of body condition (qualitative measurement of muscle and fat thickness): 1 denotes skinny, 2 denotes thin, 3 denotes ideal, 4 denotes fat, and 5 denotes obese. At the start of the experiment, the pigs were weighed and dewormed (Ivermectin, Baymec^®^ Prolong, Elanco Animal Health Korea, Songpa-gu, Seoul, Republic of Korea); two pigs were housed in each 3 × 3 m pen with a dirt floor, no roof, natural shade, sheep mesh, and electric fencing (Figure 2). The pens had individual feeders and an automatic waterer; the adaptation period to the pens was three days. The experiment was conducted in spring–summer 2017. During this period, the pigs were bathed once or twice daily during the hottest hours of the day and were weighed every 15 days.

The chemical composition of the ingredients used in the diets (Table 1) was determined at the Animal Nutrition Laboratory of the Autonomous University of Chapingo, Mexico. The experimental diets were then designed (Table 2), taking into account the protein content and the energy value. The treatments consisted of two diets, 7.79 and 13.11% crude protein, with the same metabolizable energy (3.5 Mcal/kg of dry matter). The diets were prepared based on corn, vitamins, and minerals, such that the diet with the highest protein content included soybean meal. The experimental diets were formulated according to the nutritional requirements for pigs reported by the National Research Council [15]. MCPs have lower requirements than commercial pigs, which are genetically selected to utilize large amounts of metabolizable protein and energy [16]. Water and food were provided ad libitum, the latter in two daily rations, one at 8:30 a.m. and the other at 5:30 p.m. The amount of feed consumed (kg) was determined daily using an AFK 660a scale (Adam Equipment^®^, Milton Keynes, UK) with a capacity of 300 kg and an accuracy of 20 g. The animals were weighed (kg) on an EziWeigh7i scale (Tru-Test^®^ Datamars, Lamone, Tesino, Switzerland). The feed conversion ratio (FCR) of the pigs was determined, measured as the efficiency with which pigs convert feed into body weight. The FCR was calculated by dividing the amount of feed consumed by the pig’s weight gain.

### 2.3. Slaughter and Butchering

Pigs were slaughtered when they weighed 40 and 80 kg (±3 kg). Slaughter was performed in a traditional manner following the NOM-033-SAG/ZOO-2014 standard [17], under the supervision of a veterinarian. Before slaughter, pigs fasted for 12 h. At the time of sacrifice, live weight was recorded. The pigs were desensitized by concussion using a Blitz-Kerner cattle slaughter gun; the pigs were suspended, and a cut was made in the jugular for bleeding. Viscera, head, legs, skin, and fat were removed from the carcass. The meat with bone, the skin, and the amount of back fat, defined as the layer of fat located below the skin along the back of the animal, were then weighed (g) using a scale model AFK 660a (Adam Equipment^®^, Milton Keynes, UK) with a capacity of 300 kg and precision of 20 g. The carcass was split in two until bleeding was complete (4 h). It was then refrigerated for 24 h at 4 °C [18].

The half carcasses were cut in the meat module of the Colegio Postgraduados, Veracruz campus, Mexico, to obtain primal cuts: ham, loin, shoulder, rib, and sirloin. To separate the leg, a cut was made between the second and third vertebrae of the sacral bones. From the ham, a cut was made between the second and third ribs to obtain the loin. The shoulder, aromatic and sweet, was obtained from the pig’s front legs. The rib (along with the bacon) was obtained by separating the backbone.

### 2.4. Statistical Analysis

Daily weight gain was analyzed using a mixed model:

*Y*_*ijk*lm_ = μ + D_*i*_ + S_j_ + (DS)_*i*j_ + C_k(ij)_ + A_l(ijk)_ + P_m_ + (DP)_*i*m_ + (SP)_jm_ + (DSP)_*ij*m_ + *E*_*ijk*lm_,

where

*Y_ijk_*_lm_ = Response variable;

μ = Constant characterizing the population;

D*_i_* = Fixed effect of the *i*-th diet (*i* = 1, 2);

S_j_ = Fixed effect of the j-th slaughter weight (j = 1, 2);

(DS)_*i*j_ = Fixed effect of the interaction of the *i*-th diet with the j-th slaughter weight;

*C_k(ij)_* = Random effect of the k-th nested pen in the i-th diet and j-th slaughter weight (k = 1, 2, 3, 4). $C_{k\left(ij\right)}\;\sim \;IIN\left(0,\;\sigma_{c}^{2}\right)$;

*A_l(ijk)_* = Random effect of the l-th nested pig in the i-th diet, j-th slaughter weight, and k-th pen (l = 1, 2). $A_{l\left(ijk\right)}\;\sim \;IIN\left(0,\;\sigma_{a}^{2}\right)$;

P_m_ = Effect of the m-th period (k = 1, 2, … 7, 8);

(DP)_*i*m_ = Fixed effect of the interaction of the *i*-th diet with the m-th period;

(SP)_jm_ = Fixed effect of the interaction of the j-th slaughter weight with the m-th period;

(DSP) _*ij*m_ = Fixed effect of the interaction of the *i*-th diet, the *j*-th slaughter weight, and the m-th period;

*E_ijk_*_lm_ = Experimental error. $E_{ijklm}\sim IN\left(0,\sigma_{e}^{2}\right).$

The pig, as a random effect, was eliminated for the analysis of daily feed intake and feed conversion.

A mixed model with the fixed effects of diet and slaughter weight, their interaction, and the random effect of the pen was used for the analysis of carcass and primal cut characteristics.

Data were processed using SAS 9.4 (SAS Institute Inc., Cary, NC, USA), and a comparison of means was carried out by the Tukey test (*p* < 0.05).

## 3. Results and Discussion

### 3.1. Productive Behavior

**Figure 3:**
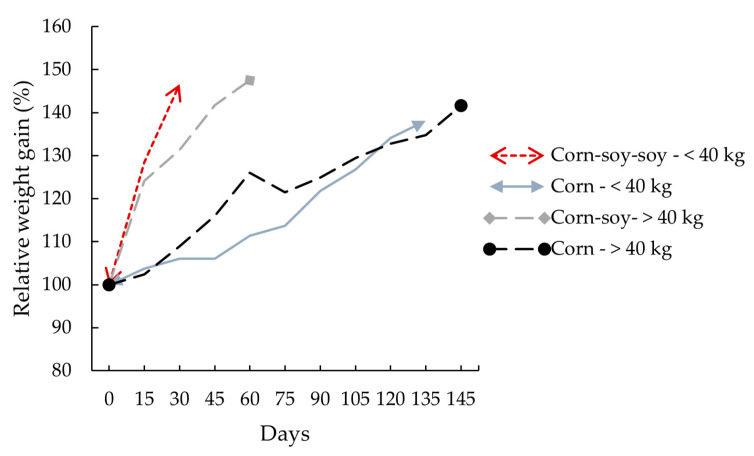
Relative weight gain and average length of stay in the pen until reaching slaughter weight in pigs fed two different diets (46% of initial weight in pigs > 40 kg and 40% of initial weight in pigs < 40 kg).

Both diet and slaughter weight were significantly associated (*p* ≤ 0.05) with respect to feed intake and weight gain (Table 3). Pigs fed the corn–soybean-based diet consumed more feed per day (approximately 495 g/d more compared with pigs fed the corn-based diet). Consequently, they gained more daily weight (160 g/d) and reached slaughter weight more quickly (Figure 3), compared with pigs fed the corn-based diet that were consequently housed longer (Figure 3).

The feed conversion ratio (FCR) did not differ significantly between diets. As expected, pigs fed the corn-based diet (DA) had a 0.8% higher FCR compared to pigs fed the corn–soybean-based diet (DB) (*p* = 0.6345). That is, they had lower feed efficiency, requiring more feed to gain weight. In young animals, the FCR is low because relative growth is high, while in older animals, the FCR is higher because growth tends to stabilize. The FCR or feed efficiency is a measure of how efficiently animals convert feed into body mass (the amount of feed consumed divided by the amount of weight gain). Thus, FCR lessens with age. Young pigs (piglets) have a very low FCR (less than 1) because of the rapid development of muscle and bone. As pigs mature, the FCR gradually changes because more fat is deposited, which requires more energy from feed. Thus, at slaughter, the FCR is around 3.0 [19]. Thus, the feed rate and meal size do not have as much influence on feed efficiency as age [20]. In the present study, the FCR was almost double the value reported for adult pigs before slaughter (FCR of 3.0). This means that the animal requires more feed to gain body mass, resulting in a low efficiency in terms of feed conversion. This behavior is partly due to the fact that the pigs used in this study were adults (over 8 months of age). Thus, in the study conducted by Montiel-Pérez et al. [21], a feed conversion ratio between 2.83 and 3.48 was obtained in young Mexican hairless pigs (initial weight of 12 kg and slaughter weight between 23 and 27 kg) fed with diets rich in lysine, with daily weight gains similar (0.22–0.31 kg/day) to those of the present investigation. On the other hand, the MCP is slow growing and presents an obese genotype, such that the deposition of adipose tissue increases and that of muscle mass becomes constant, decreasing the efficiency of feed conversion (i.e., a higher FCR) [22]. In terms of energy, the formation of adipose tissue is much more expensive in terms of foodstuff than the formation of muscle mass. Thus, Ramon-Canché et al. [4] found that, during the growth period of MCPs (20–25 kg), a high daily weight gain (336 g) and lower feed consumption can be observed. In this way, growing pigs can achieve better feed conversion ratios (2.92 kg:1 kg). Body fat deposition in MCPs is associated with obesity-related gene expression rather than increased feed intake [23]. Unlike MCPs, commercial pigs exhibit greater weight gain and feed intake. Thus, the nutritional requirements of MCPs are different, as are growth rate, body composition, and weight gain composition, due to the high rate of lipogenesis (greater backfat thickness).

Peralta et al. [24] found that diet has a significant effect on the consumption pattern of MCP; a diet with good palatability and rich in fat encourages pigs to consume and utilize it better. Furthermore, the chemical composition and nutritive value of commercial soy products vary according to the source and commercial batch, with a significant effect on growth and nutrient digestibility in piglets [25]. These researchers found that US soybean meal improves average daily gain, gain-to-feed ratio, and nutrient digestibility compared to South American meal. In accordance with Ramos-Canché et al. [4], the nutritional requirements of MCPs are very different from the growth models published for lean genotypes [11]. Females consuming feed with 3200 kcal of metabolizable energy/kg had higher daily weight gain and reached the desired slaughter weight in a shorter time with lower feed intake and better feed conversion. Pigs fed corn–soybean reached slaughter weight sooner, probably because the crude protein content in this diet is twice as high as the corn-based diet. Another determining factor in this performance is the feeding system (individual feeders in pens, ad libitum), which significantly influences the weight gain of MCP.

Productive responses across both slaughter weights indicate that MCPs slaughtered at 80 kg live weight consumed more feed and gained more weight (1 kg/d and 160 g/d, respectively) compared to pigs slaughtered at 40 kg (Table 3). Pigs slaughtered at 40 kg had significantly higher FCR and were less efficient in terms of gaining weight. The productive performance of MCPs is quite variable because many factors influence weight gain, feed intake, and FCR. For example, climate, feeding system, age, animal history, diet (nutrient content and calorific value), slaughter weight, and diet duration make it difficult to compare our results with those of other researchers in order to make practical recommendations. Lemus et al. [22] observed that pigs raised in a semi-warm environment (as in the present investigation) and without grazing have a higher growth rate (weight/d) and higher final weight than those raised in a warm climate. Regarding the slaughter weight, Becerril et al. [26] found that MCPs slaughtered at 83 kg of live weight and fed with a commercial diet consumed 1.77 kg/d of feed and gained 414 g/d of weight, such that the FCR was 4.27, a lower value than the one obtained in the present investigation. Similarly, in pigs with less live weight (20–35 kg) and older than one year of age, Sierra-Vásquez et al. [27] and Ortiz et al. [28] reported feed intakes of 1.25 kg/animal/day and weight gains of 345 g/animal/day, achieving better feed conversion (3.75–3.83) using comprehensive forage-based diets with 15% crude protein.

### 3.2. Carcass Performance

Both diets produced the same effect on the yield of the head, the entire carcass (without head, kidney, and diaphragm), and its components (meat, fat, and skin) (*p* = 0.4355). Carcass yield was less than 70%, whereas including the head, the yield was 76–80%. These values have been observed in commercial pigs [29]. On average, the carcass components (meat, fat, and skin) presented proportions of 60%, 30%, and 10%, respectively (Table 4).

Animals slaughtered at a higher live weight had significantly lower head yield and higher fat yield (*p* < 0.05), with meat yield decreasing by up to 7% compared to pigs slaughtered at 40 kg (Table 4). The accumulation of fat in MCPs is a characteristic that makes them less preferred by commercial lean meat producers and meat marketers. Although it has been documented that decreasing the protein–energy ratio of the diet produces fattier carcasses [30], in the present study, the decrease in protein content (lower protein–energy ratio) had no effect on carcass fat content. According to Lebret [30], the protein–energy ratio can be used to manipulate growth rate and weight gain composition. Decreasing the protein–energy ratio of the diet (amino acid-deficient diet) decreases growth rate and increases interstitial fat deposition and, to a lesser extent, backfat thickness. Mexican hairless Creole pigs (MCPs) are a local breed known for their higher fat content compared to commercial breeds [12]. Therefore, in the present investigation, the protein content in the evaluated diets was not sufficient to modify the backfat content in MCP, since both evaluated diets had the same amount of metabolizable energy. When there is a limited energy allocation, backfat presents minimal changes but increases the growth-fattening period, the age at slaughter, and intramuscular fat deposition [31]. In terms of age, intramuscular fat deposition develops later in fatty genotypes compared to other fat depots [32], such that backfat only “moderates” when the pigs are adults. This is why significant differences in backfat content due to the effect of initial weight were observed in the present study. It is likely that both the genotype and its interaction with age influence fat deposition after 40 kg of live weight. The level of dietary protein in the diet is more closely related to fat composition (presence of specific fatty acids and amino acids), growth performance, and intramuscular fat than to the amount of subcutaneous fat [33].

Other studies have reported carcass yields quite similar to those obtained in the present study. Becerril et al. [34] reported slightly lower carcass yields (62.7%) in pigs slaughtered with a live weight greater than 100 kg. On the other hand, Ortiz et al. [21] obtained higher carcass yields (71.10%) when MCPs were slaughtered at 40 kg live weight. Similar yields were also reported by Santos et al. [35], who found a carcass yield of 77.8%. Low carcass yield is associated with poor body development when pigs have a diet insufficient in nutritional requirements. On the other hand, high fat content seems to be related to the genotype and older age of the animals [36,37,38]. Pigs slaughtered at 80 kg live weight had significantly higher fat percentages than those slaughtered at 40 kg because they spent more time in the pen and were older, regardless of the type of diet (Figure 2). Thus, Virgili et al. [39] found that carcasses from 10-month-old pigs had lower muscle mass indexes and lean cut yields than those from younger pigs (8 months). Furthermore, carcasses from faster-growing pigs tended to be fattier than those from slower-growing ones [40]. However, in the present study, diet had no effect on carcass fat content, even though pigs fed corn–soybean reached slaughter weight more quickly.

The increase in backfat in MCPs is due to the fact that they tend to deposit more fat in the carcass than commercial pigs. Backfat is usually expressed as backfat thickness and is measured at the midline of the right half of the carcass [41], so there are no studies that determine the amount of backfat (kg) in carcasses. However, the MCP is a descendant of the Iberian pig introduced during the Spanish conquest, so it is characterized by having a high deposition of both dorsal and interstitial (intramuscular) fat. According to Santos et al. [42], this particularity is due to the fact that MCPs have not undergone any genetic improvement to increase their muscle tissue yield. Although fat accumulation is directly associated with weight and age, backfat in MCPs is greater than in commercial pigs. Thus, Méndez et al. [43] reported total intramuscular fat contents of 40.7% in the loin of castrated MCPs of 115 kg live weight at slaughter fed with commercial concentrate, while in commercial pigs (Duroc × Landrace × Yorkshire), Correa et al. [40] reported loin intramuscular fat contents of only 31.7% in castrated males of 115 kg. In both studies, fat extraction was performed with solvents.

### 3.3. Weight and Yield of Primal Cuts

Our results show that sirloin yield was significantly higher using the corn–soybean-based diet. Since sirloin has a tender texture and is a lean, juicy cut of meat, being a muscle located below the loin (it does not bear much weight or involve much movement), it is commercially important for roasting and for making steaks. The yield of the other primal cuts did not differ due to the effect of diet. However, yield was relatively higher when the corn–soybean-based diet was used (Table 5), so this diet can be recommended in MCPs to improve meat yield.

Regarding slaughter weight, the results showed that ham yield was significantly lower when pigs were slaughtered at 80 kg live weight. Consequently, under the experimental conditions of our study, regardless of the type of diet (corn or corn–soy) and under a semi-technological production system, we can recommend slaughtering MCPs at 40 kg of live weight when seeking better meat yields, since the ham or leg is the primal cut with the highest proportion compared to the other primal cuts, and is commercially relevant in the cured products industry. There is a direct proportional relationship between weight at slaughter and live weight gain and carcass weight in pigs. However, backfat depth also tends to increase, as does the fat content of the half carcass and the primal cuts in the form of shoulder, loin, belly, and ham [44]. In commercial pigs, the goal is to improve intramuscular fat but not the development of subcutaneous and intermuscular fat deposition. Dietary modifications during the growing phase appear to increase intramuscular fat deposition [44]. Yields of primal cuts in MCPs are usually quite low compared to those of commercial pigs. In ham, for example, Iberian × Duroc pigs have a yield between 22.9% and 23.3% compared to MCPs (15.4–18.2%) [45]. The averages of the primal cuts (kg) obtained in the present investigation (Table 6) are higher than those obtained by other researchers with regard to MCPs but lower than those in commercial pigs. In Large White × Landrace × Duroc pigs, D’Souza et al. [44] reported an increase in loin weights from 4.08 to 7.47 kg depending on the age at slaughter (16 or 25 weeks). In the present investigation, loin weight was only 0.7–1.19 kg in MCPs of comparable ages (6–24 weeks old). The trend is quite similar for the other primal cuts. Even lower loin weights have been obtained in studies conducted with MCPs. Sierra-Vásquez et al. [27] reported loin weights ranging from 0.59 to 0.81 kg during moringa flour supplementation in integral diets of 20–35 kg live weight pigs (15% protein) fed for 105 days. According to Santos et al. [35], the highest growth rate with regard to legs and shoulders in MCPs with respect to live weight is observed between 37 and 42 kg, such that the yield of primal cuts and carcass fat increases as slaughter weight increases. These researchers reported increasing loin weights from 2.3 to 5.2 kg in pigs slaughtered at 25–45 kg live weight (entire carcass). The results of the present study and those of Sierra-Vásquez et al. [27] considered the loin weight of the half carcass; therefore, the weight of the loin (kg) was lower. Furthermore, in the study conducted by Santos et al. [35] with a diet formulated to provide 18% crude protein and 3300 kcal of ME/kg of dry matter and made with a base of sorghum and soybean paste, requirements were higher than those of the present investigation.

## 4. Conclusions

Replacing corn with soybeans in diets to increase protein levels can be effective for feeding tropical Mexican hairless Creole pigs, as it increases feed intake and weight gain. Additionally, incorporating soybeans into diets can reduce pen time. While the use of soybeans in the diet increases the amount of sirloin, live weight at slaughter has a greater influence on carcass and primal cut yield. A higher slaughter weight increases backfat yield and the amount (kg) of primal cuts but reduces head and ham (leg) yield. The use of soybeans in the diet and slaughter at higher live weights can be recommended because they allow for the preservation of traditional regional diets by using carcasses with a high backfat content.

## Figures and Tables

**Table 1:** Chemical composition of the ingredients (% dry matter basis) used in the experimental diets.

Fraction Analysis	Ingredient	
	Corn (%)	Soy (%)
Dry matter	85.40	89.80
Crude protein	8.07	44.70
Ether extract	0.38	1.03
Ash	1.90	7.20
Crude fiber	3.37	7.30

**Table 2:** Design of experimental diets (% dry basis) for feeding Mexican Creole pigs (MCPs).

Ingredient	Diet	
	DA (%)	DB (%)
Soybean paste	-	14.50
Ground yellow corn	96.50	82.00
Vitamins	1.00	1.00
Minerals	2.50	2.50
Chemical Composition		
Dry Matter (DM)	85.40	86.90
Protein (%)	7.79	13.11
Metabolizable Energy (Mcal/kg DM)	3.50	3.50

DA: Diet 1, lower protein content; DB: Diet 2, higher protein content.

**Table 3:** Effect of diet and slaughter weight on the productive characteristics of Mexican Creole pigs (MCPs).

Factor	Feed Consumption (kg/d)	Weight Gain (kg/d)	Feed Conversion Ratio
Diet			
DA	1.33 ± 0.11 ^b*^	0.17 ± 0.03 ^b*^	6.20 ± 0.56 ^a^
DB	1.83 ± 0.12 ^a*^	0.33 ± 0.03 ^a*^	5.40 ± 0.63 ^a^
Weight (kg)	Feed consumption (kg/d)	Weight gain (kg/d)	Feed conversion ratio
40	1.21 ± 0.12 ^b**^	0.17 ± 0.03 ^b*^	6.00 ± 0.61 ^a^
80	2.00 ± 0.11 ^a**^	0.33 ± 0.03 ^a*^	5.20 ± 0.58 ^a^

* Different letters in the same column indicate significant statistical differences (Tukey’s multiple range tests, assuming a significant difference at *p* < 0.05); ** (*p* ≤ 0.01). DA = Corn; DB = Corn–Soy.

**Table 4:** Effect of diet and slaughter weight on carcass yield (%) of Mexican Creole pigs (MCPs) fed corn and corn–soybean-based diets.

Factor	Yield (%) ^x^		Yield (%), Headless Carcass		
Diet	Carcass	Head	Meat	Fat	Skin
DA	69.50 ± 1.2 ^a^	10.70 ± 0.60 ^a^	59.60 ± 2.20 ^a^	30.50 ± 2.10 ^a^	10.00 ± 1.00 ^a^
DB	66.80 ± 1.3 ^a^	9.20 ± 0.60 ^a^	61.70 ± 2.40 ^a^	29.30 ± 2.30 ^a^	8.20 ± 1.00 ^a^
Weight (kg)	Carcass	Head	Meat	Fat	Skin
40	68.00 ± 1.2 ^a^	11.50 ± 0.60 ^a**^	64.20 ± 2.20 ^a^	25.10 ± 2.10 ^b*^	10.10 ± 1.00 ^a^
80	68.00 ± 1.3 ^a^	8.50 ± 0.60 ^b**^	57.10 ± 2.40 ^a^	34.60 ± 2.30 ^a*^	8.00 ± 1.00 ^a^

* Different letters in the same column indicate statistically significant differences (Tukey’s multiple range tests, assuming a significant difference at *p* < 0.05); ** (*p* ≤ 0.01). ^x^ Performance relative to live weight. DA = Corn; DB = Corn–Soy.

**Table 5:** Effect of diet and slaughter weight on the yield of primal cuts (%) of Mexican Creole pigs (MCPs) fed corn and corn–soybean-based diets.

Factor	Yield (%)				
Diet	Ham ^X^	Shoulder ^X^	Loin ^X^	Rib ^X^	Sirloin ^X^
DA	15.40 ± 0.80 ^a^	12.00 ± 0.96 ^a^	4.30 ± 0.34 ^a^	12.00 ± 0.58 ^a^	0.59 ± 0.04 ^b*^
DB	18.20 ± 0.85 ^a^	14.30 ± 1.00 ^a^	4.90 ± 0.37 ^a^	12.60 ± 0.62 ^a^	0.80 ± 0.05 ^a*^
Weight (kg)	Ham ^X^	Shoulder ^X^	Loin ^X^	Rib ^X^	Sirloin ^X^
40	18.10 ± 0.79 ^a*^	13.00 ± 0.96 ^a^	4.9 ± 0.34 ^a^	11.70 ± 0.58 ^a^	0.72 ± 0.04 ^a^
80	15.40 ± 0.85 ^b*^	13.30 ± 1.00 ^a^	4.3 ± 0.37 ^a^	12.80 ± 0.62 ^a^	0.67 ± 0.05 ^a^

* Different letters in the same column indicate significant statistical differences (Tukey’s multiple range tests, assuming a significant difference at *p* < 0.05); DA = Corn; DB = Corn–Soy. ^X^: right half carcass.

**Table 6:** Effect of diet and slaughter weight on the weight of primal cuts (kg) of Mexican Creole pigs (MCPs) fed corn- and corn–soybean-based diets.

Factor	Weight (kg)				
Diet	Ham ^X^	Shoulder ^X^	Loin ^X^	Rib ^X^	Sirloin ^X^
DA	3.20 ± 0.27 ^a^	2.50 ± 0.21 ^a^	1.00 ± 0.08 ^a^	2.50 ± 0.16 ^a^	0.13 ± 0.12 ^a^
DB	3.60 ± 0.30 ^a^	3.00 ± 0.22 ^a^	1.00 ± 0.08 ^a^	2.6 ± 0.17 ^a^	0.16 ± 0.13 ^a^
Weight (kg)	Ham ^X^	Shoulder ^X^	Loin ^X^	Rib ^X^	Sirloin ^X^
40	2.50 ± 0.27 ^b*^	1.80 ± 0.21 ^b*^	0.70 ± 0.08 ^b*^	1.60 ± 0.15 ^b*^	0.10 ± 0.12 ^b*^
80	4.30 ± 0.30 ^a*^	3.60 ± 0.22 ^a*^	1.19 ± 0.90 ^a*^	3.50 ± 0.17 ^a*^	0.18 ± 0.13 ^a*^

* Different letters in the same column indicate significant statistical differences (Tukey’s multiple range tests, assuming a significant difference at *p* < 0.01); DA = Corn; DB = Corn–Soy. ^X^: right half carcass.

## Data Availability

The dataset is available upon request from the authors.
